# Independent evolution of pain insensitivity in African mole-rats: origins and mechanisms

**DOI:** 10.1007/s00359-020-01414-w

**Published:** 2020-03-23

**Authors:** Ewan St. John Smith, Thomas J. Park, Gary R. Lewin

**Affiliations:** 1 Department of Pharmacology, University of Cambridge, Cambridge, CB2 1PD UK; 2 Laboratory of Integrative Neuroscience, Department of Biological Sciences, University of Illinois at Chicago, Chicago, IL USA; 3 Molecular Physiology of Somatic Sensation, Max Delbrück Center for Molecular Medicine, Robert-Rössle Str. 10, D-13125 Berlin, Germany

**Keywords:** Evolution, pain, nociception, ion channels, African mole-rats

## Abstract

The naked mole-rat (*Heterocephalus glaber*) is famous for its longevity and unusual physiology. This eusocial species that lives in highly ordered and hierarchical colonies with a single breeding queen, also discovered secrets enabling somewhat pain-free living around 20 million years ago. Unlike most mammals, naked mole-rats do not feel the burn of chili pepper’s active ingredient, capsaicin, nor the sting of acid. Indeed, by accumulating mutations in genes encoding proteins that are only now being exploited as targets for new pain therapies (the nerve growth factor receptor TrkA and voltage-gated sodium channel, Na_V_1.7), this species mastered the art of analgesia before humans evolved. Recently, we have identified pain insensitivity as a trait shared by several closely related African mole-rat species. One of these African mole-rats, the Highveld mole-rat (*Cryptomys hottentotus pretoriae*), is uniquely completely impervious and pain free when confronted with electrophilic compounds that activate the TRPA1 ion channel. The Highveld mole-rat has evolved a biophysical mechanism to shut down the activation of sensory neurons that drive pain. In this review, we will show how mole-rats have evolved pain insensitivity as well as discussing what the proximate factors may have been that led to the evolution of pain-free traits.

## Introduction

Pain is so central to our personal experience that we as humans assume that all non-human animals experience pain in a similar way to ourselves. Indeed, when we observe rodents that are injured, they exhibit behaviors and even facial expressions (Langford et al. 2010) that give us the feeling that their pain experience is similar, if not identical to ours. Indeed, it makes sense that pain should be a universal mechanism that enables all animals to avoid injury as well as contact with things and substances that can harm the organism. The universality of nociception (Smith and Lewin 2009) makes it all the more surprising when one encounters absence of pain behavior in distinct species. In this review we will concentrate on some recently described instances of pain insensitivity in vertebrates. We will discuss the mechanisms underlying loss of pain sensation, but also examine the possible proximate causes that may have led to the evolution of distinct pain insensitivities.

Examining pain-related behavior is a challenging task even in conventional laboratory rodent models like *Mus musculus* and *Rattus rattus*. Typically, pain behaviors are assessed by measuring reflex withdrawal from a noxious stimulus which may be radiant heat, mechanical force or cold. Such parameters are hard to measure and compare between different species. However, it has long been known that naturally occurring chemical substances, called algogens evoke painful sensations or avoidance in animals and humans. One of the best characterized algogens is capsaicin, a natural substance and the active ingredient of chili peppers, and the substance that confers the burn of hot peppers (Caterina et al. 2000; Caterina and Julius 2001). There is a huge literature on capsaicin, characterizing its neurotoxic effects in neonatal animals, as well as its use as a substance to produce acute experimental pain in humans (Jancsó et al. 1977; Treede et al. 1992). Capsaicin causes burning sensations in humans and is thought to induce a similar sensation in most vertebrates, one major exception being all birds, which have evolved insensitivity to capsaicin as they are seed carriers for capsicum plants (Jordt and Julius 2002). It is not clear when capsaicin insensitivity evolved in the bird lineage, but it is possible that the ancestors of birds, i.e., non-avian dinosaurs, were already insensitive to capsaicin. In 2008, we discovered that capsaicin was not a universal irritant in mammals as our studies on the nociceptive system of the naked mole-rat (*Heterocephalus glaber*) revealed that this unusual subterranean mammal was completely insensitive to capsaicin (Park et al. 2008). Naked mole-rats have fascinated biologists for several decades as this underground dwelling species native to East Africa has a unique biology. Naked mole-rats are one of only two eusocial mammals that live in very large colonies (up to 300 individuals) with a single breeding female who is the queen (Jarvis 1981; Jarvis et al. 1994). In the late 1970s Dr Jenny Jarvis from the University of Cape Town was the first to show that these animals could be kept and bred in the laboratory, and today naked mole-rat colonies are maintained in many labs worldwide. Naked mole-rats have so many extreme physiological features that we would need an entire review to detail them all. Some of their most striking physiological adaptations include an extraordinarily long maximum life span of at least 36 years (Ruby et al. 2018), apparent resistance to cancer, and resistance to extreme hypercapnia and hypoxia (Park et al. 2017). In this review we will examine the questions of how and why naked mole-rats lost certain types of pain sensitivity. Since the naked mole-rat is a member of a very large group of underground dwelling African rodents we will also examine if pain insensitivity is a family affair.

## The nociceptive system: anatomy and physiology

The nociceptive system is highly conserved and any deficit in pain sensitivity could in principle be linked to molecular changes herein. The experience of pain requires that specialized sensory neurons that are the primary sensors of the nociceptive system are activated. Sensory neurons that detect potentially damaging and painful stimuli have their cell bodies in the dorsal root ganglia (DRG) and co-exist with other sensory neurons specialized to detect innocuous light touch and non-noxious thermal stimuli (Heppenstall and Lewin 2000; Lewin and Moshourab 2004; Dubin and Patapoutian 2010; Lechner and Lewin 2013; Lewin et al. 2014; Smith 2018). Anatomically, sensory neurons have been primarily classified according to the size and myelination state of the peripheral axon. Sensory neurons that mediate the sensation of touch have large diameter Aβ-fibers that are thickly myelinated and some medium diameter thinly myelinated Aδ-fibers, called D-hair receptors (Wang and Lewin 2011; Heidenreich et al. 2012; Lechner and Lewin 2013). A larger group of sensory neurons with thinly myelinated Aδ-fibers are so-called nociceptors and alongside unmyelinated C-fibers are the primary mediators of the painful sensation (Lewin and Moshourab 2004; Smith and Lewin 2009). The cell bodies of those sensory neurons innervating most of the body are located in the DRG, but those innervating the head are located in the trigeminal ganglia (TG). Nociceptive sensory neurons can detect and signal a wide variety of different stimuli including heat, cold, mechanical and chemical stimuli. Indeed most single nociceptors respond to more than one type of noxious stimulus and are often classified as polymodal, a fact that has been repeatedly confirmed over several decades (Bessou and Perl 1969; Lewin and Mendell 1994; Perl 1996; Milenkovic et al. 2008; Smith and Lewin 2009). Recent molecular analyses of nociceptive populations have highlighted the diversity of molecular receptors that are expressed by individual nociceptors (Usoskin et al. 2015; Li et al. 2016; Hockley et al. 2019).

## The nociceptive system in naked mole-rats

The first detailed examination of sensory anatomy in the naked mole-rat demonstrated that the skin of this largely hairless species is innervated by both myelinated and unmyelinated fibers (Park et al. 2003). In most rodents unmyelinated fibers can be subdivided into peptidergic and non-peptidergic fibers based upon their expression of neuropeptide neurotransmitters such as calcitonin gene-related peptide (CGRP) and substance P (SP) (Nagy and Hunt 1982; Silverman and Kruger 1990; Stucky and Lewin 1999). However, in the naked mole-rat, unmyelinated fibers in the skin were observed to be virtually devoid of SP and CGRP immunostaining; CGRP immunostaining was, however, observed in lanceolate endings that surround hair follicles, which probably arose from Aβ-fibers. When Park and colleagues examined the neuropeptide immunoreactivity in DRG and TG, unlike in normal lab rats (*Rattus rattus*), intense CGRP and SP immunoreactivity were essentially absent from small diameter (i.e., likely nociceptive) neurons. However, strong CGRP staining was observed in some large diameter neurons, which correlated with the staining of lanceolate endings observed in the skin (Park et al. 2003). In addition, naked mole-rats have visceral fibers that are also positive for CGRP (Park et al. 2003; Hockley et al. 2020).

The lack of neuropeptide-positive sensory neurons in the naked mole-rat raised the question of whether this species had lost this sensory population in the course of evolution. One way to examine such a question is to quantify the numbers of unmyelinated axons in peripheral nerves which is normally done with transmission electron microscopy. The anatomy of the saphenous and sural nerves have been examined in a wide range of species as these nerves innervate hindlimb hairy skin and contain almost exclusively cutaneous axons (Lewin and McMahon 1991). Many groups have quantified the numbers of myelinated (A-fibers) and unmyelinated (C-fibers) and consistently observed that there are always 3–4 times as many C-fibers as A-fibers in these nerves (Ochoa and Mair 1969; Alpson and Lal 1980; Scadding 1980; Schwab et al. 1984; Illanes et al. 1990; Stucky et al. 2002; Wetzel et al. 2007; Milenkovic et al. 2007; Stürzebecher et al. 2010). However, in naked mole-rat, this ratio is just ~ 1.5:1 and the low ratio is due to absolute paucity of unmyelinated nociceptors and not due to increased numbers of A-fiber axons (Park et al. 2008; Smith et al. 2012; Omerbašić et al. 2016). Thus, naked mole-rats have far fewer cutaneous nociceptors than other similarly sized mammals, which prompted us to study-related subterranean mole-rat species that are phylogenetic neighbors to the naked mole-rat. In a comparative study of nerves from six species belonging to the Bathyergidae, the naked mole-rat was the only species with markedly low cutaneous C to A-fiber ratio (Smith et al. 2012). An analysis of the A- and C-fiber numbers related to surface area determined that this was accounted for by a paucity of C-fibers, rather than an overabundance of A-fibers (Smith et al. 2012). It is likely that mammals have evolved high density cutaneous nociceptor innervation as the skin is constantly exposed to potentially damaging stimuli. Consistent with this idea, nerves that innervate muscle and joints do not display high ratios of C- to A-fibers as is seen in the skin and this is true of all mammals examined. Indeed, all bathyergids, including the naked mole-rat, and a wide range of mammals typically display a ~ 1:1 C- to A-fiber ratio in muscle nerves (Jenq et al. 1984; Jenq and Coggeshall 1984, 1985; Smith et al. 2012). A potentially parsimonious explanation for the cutaneous C-fiber deficit in naked mole-rats is that they largely lack fur, although specialized sensory hairs are present (Crish et al. 2003). Compared to most other mammals, humans are relatively naked and lack dense body hair; however, human cutaneous nerves also show a ~ 4:1 C to A-fiber ratio (Ochoa and Mair 1969). To our knowledge no such investigation has yet been conducted in other “naked” mammals, such as the manatee (*Trichechus manatus*) or hairless bat (*Cheiromeles torquatus*). We instead examined cutaneous nerves in transgenic mice that are born with hair, but lose hair follicles and thus partially resemble naked mole-rats, but these animals display only a mild decrease in C- to A-fiber ratio indicating that loss of hair follicles cannot explain the substantial loss of C-fibers in the naked mole-rat (Smith et al. 2012); indeed, initial analysis showed that the naked mole-rat epidermis is more densely innervated than that of the rat (*R. norvegicus*) and common mole-rat (*Cryptomys hottentotus hottentotus*) suggesting that in the absence of hair follicles sensory axons innervate other structures in the skin (Park et al. 2003). In normal mice it has been observed that there is ongoing naturally occurring cell death of sensory neurons with C-fiber axons in the early postnatal period. This loss of axons is reflected in a drop in the C- to A-fiber ratio from ~ 6:1 to ~ 4:1 in adults. We recently observed a similar, but more extreme, process in naked mole-rats, such that postnatal day 3 naked mole-rat pups have a C- to A-fiber ratio of ~ 6:1 that drops to ~ 1:1 in adults (Omerbašić et al. 2016). The factors that might cause nociceptor loss is discussed below in relation to the biology of nerve growth factor (NGF) and its receptor tropomyosin receptor kinase A (TrkA).

## Nociceptor function and nocifensive behaviors in naked mole-rats

Transgenic mice that lack CGRP and SP show diminished pain behaviors (Cao et al. 1998; Salmon et al. 2001) and thus the absence of neuropeptides in naked mole-rat nociceptors raised the question of whether or not naked mole-rat nociceptors function as in other rodents. This observation also prompted us to ask whether naked mole-rats display the same repertoire of nocifensive behaviors as observed in other rodents. The existence of nociceptors is almost completely ubiquitous in the animal kingdom and even the nematode worm *Caenorhabditis elegans*, with approximately 300 neurons, has a dedicated set of nociceptors (Tobin and Bargmann 2004; Smith and Lewin 2009), the fundamental importance of nociceptors is demonstrated by humans with congenital insensitivity to pain (CIP), a syndrome that can arise due to a complete absence of nociceptive Aδ- and C-fibers (Swanson et al. 1965; Rafel et al. 1980). The loss of nociceptors observed in some individuals with CIP is chiefly due to mutations in nerve growth factor (NGF) or its receptor TrkA (Indo et al. 1996), indeed mice in which the gene encoding the NGF receptor TrkA also lack C-fibers (Smeyne et al. 1994). Congenital insensitivity to pain and nociceptor loss is also associated with mutations in other genes for example PRDM12 (PRDI-BF1 and RIZ homology domain-containing protein 12) (Chen et al. 2015).

Despite the fact that cutaneous nerves in naked mole-rats have a depleted number of C-fiber nociceptors, our physiological studies have shown that the receptor properties of these neurons do not differ significantly from C-fibers in mice (Park et al. 2008). This was the case for saphenous nerve Aδ-fibers with nociceptive properties, as well as for C-fiber nociceptors as measured using an ex vivo skin–nerve preparation (Milenkovic et al. 2008; Moshourab et al. 2013; Walcher et al. 2018). The major C-fiber nociceptor population is classified as polymodal receptors (i.e., responding both to mechanical and thermal stimuli) and they make up the majority of C-fibers (60–70%) (Fleischer et al. 1983; Lewin and Mendell 1994; Koltzenburg et al. 1997; Lewin and Moshourab 2004). The second population are C-fibers, which are activated only by intense mechanical stimuli, and which make up the remaining functional population and it is these fibers that appear to have different mechanosensitive properties compared to polymodal C-fibers (Milenkovic et al. 2008; Bishop et al. 2010). Interestingly, these two C-fiber populations were present in naked mole-rats with receptor properties that were almost identical to those of other rodents (Park et al. 2008). Thus, the loss of C-fibers in the naked mole-rat does not appear to be due to loss of one functional sub-population and the lack of neuropeptides in these C-fibers appears to be unrelated to their receptive properties.

**Fig. 1:**
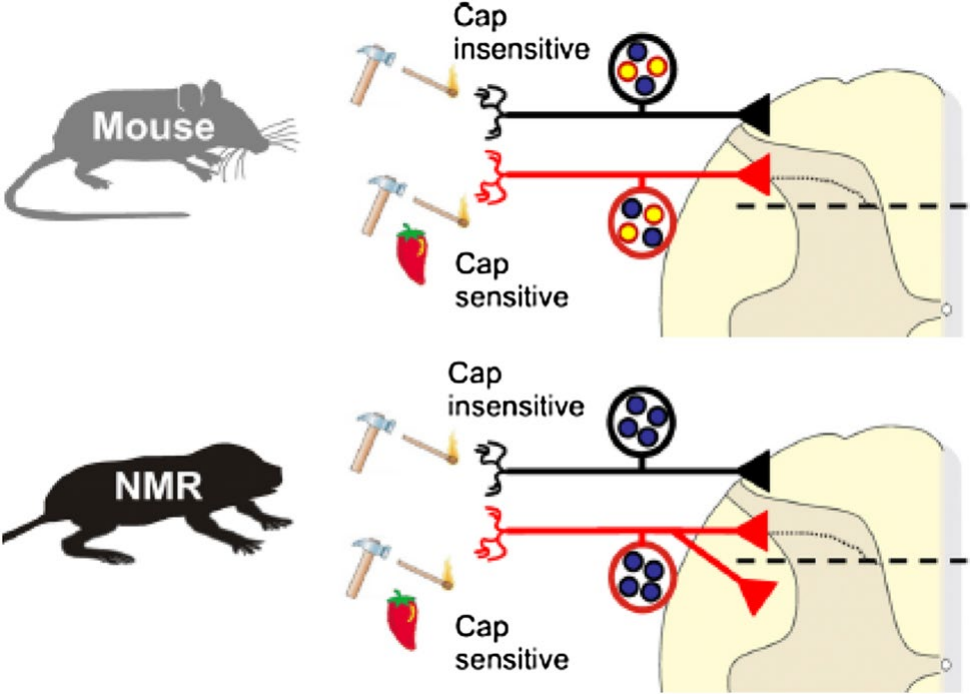
Altered nociceptive circuitry in the naked mole-rat. Functional and anatomical data suggest that TRPV1 positive nociceptors in the naked mole-rat make synaptic connections with both superficial and deep dorsal horn neurons. Yellow circles represent peptidergic releasing C-fibers, blue circles represent C-fibers only releasing glutamate-containing vesicles. This altered connectivity compared to all other species could be one reason why naked mole-rats show no behavioral response to capsaicin (Figure reproduced from Park et al 2008)

Nocifensive behaviors, which are often measured as rapid limb withdrawal from a mechanical or thermal stimulus were broadly similar between mouse and naked mole-rat (Park et al. 2008). However, it should be noted that it is almost impossible to use the same kinds of behavioral testing and conditions to directly compare thresholds across species. Comparisons of behavioral responses to innocuous mechanical stimuli have been made using the Schallert tape removal test (time taken to remove a square of adhesive tape place on a forepaw) and honey test (time taken to wipe the face in response to a drop of runny honey dropped on to the face between snout and eyes) and here naked mole-rats displayed significantly longer latency times than mice (Deacon et al. 2012). The behavioral significance or relevance of these behaviors to the ecological niche of the naked mole-rat are, however, unclear.

Naked mole-rats show rapid paw withdrawal from a radiant heat source which is consistent with our observations that the naked mole-rat is equipped with noxious heat sensitive C-fiber nociceptors (Park et al. 2008). Withdrawal from noxious heat measured using the Hargreaves test (Hargreaves et al. 1988; Deuis et al. 2017) is often used to assess changes or modulations of pain behavior, e.g., increased sensitivity to pain following injury. Some studies have evaluated how different compounds modulate naked mole-rat behavioral sensitivity to noxious heat. Unexpectedly, morphine caused the latency to decrease (i.e., increased sensitivity/pain), which was suggested to be due to causing motor hyperexcitability and when group housed, naked mole-rats exposed to morphine became aggressive, attacking and killing each other (Kanui and Hole 1990). A similar, pain sensitizing effect was observed in naked mole-rats with pethidine in a hot plate test, pethidine is, like morphine, a non-selective opioid receptor agonist and the effects were prevented by co-administration of the non-selective opioid receptor antagonist naloxone (Towett and Kanui 1993). More recent analysis using selective agonists has shown that activation of both mu and delta opioid receptors using DAMGO and DPDPE respectively, causes hyperalgesia in the hot plate test that was reversed by naloxone and by naloxonazine (the latter a mu opioid receptor antagonist). In contrast, kappa-receptor agonists (U-50488 and U-69593) caused anti-nociceptive effects in the same assays (Towett et al. 2006). In contrast to morphine, nefopam (an inhibitor of monoamine reuptake transporters) produced a dose-related increase in nociceptive latency (Kanui and Hole 1990). Lastly, aspirin and indomethacin, both inhibitors of cyclooxygenase enzymes and thus prostaglandin production, produce analgesia in the hot plate test in naked mole-rats (Towett and Kanui 1993).

The differences in nocifensive behaviors between other rodents and the naked mole-rat were more dramatic when we examined their behavioral response to algogens. Algogens are chemicals that directly produce pain sensation in humans and animals and often work by directly activating specific ion channels or receptors expressed by nociceptors, thereby potently activating sensory receptors that initiate pain. Capsaicin is a prototypical algogen and is the chemical in chili peppers of the genus Capsicum that gives rise to the sensation of heat experienced when eating food flavored with chilies. When injected into mice, capsaicin evokes a nocifensive licking response, which is due to its specific activation of the transient receptor potential vanilloid 1 channel (TRPV1), a heat-, capsaicin- and acid-gated ion channel expressed by nociceptors (Caterina et al. 1997, 2000; Tominaga et al. 1998). Capsaicin robustly activates naked mole-rat polymodal C-fibers as determined by direct electrophysiological recordings with the ex vivo skin–nerve preparation and patch clamp recordings from isolated DRG neurons (Park et al. 2008; Smith et al. 2011; Omerbašić et al. 2016). However, despite the fact naked mole-rat nociceptors are activated by capsaicin, foot pad injection of capsaicin fails to evoke any licking behavior in naked mole-rats (Park et al. 2008; Eigenbrod et al. 2019). In addition, whereas administration of capsaicin causes sensitization of the response to heat (thermal hyperalgesia), this phenomenon also does not occur in naked mole-rats (Park et al. 2008). How then is it that capsaicin activation of nociceptors does not lead to pain behavior? The majority of nociceptive input to the spinal cord terminates in the superficial dorsal horn (Todd 2010, 2017) and because DRG neurons are pseudounipolar, proteins that are expressed at the peripheral terminal are also commonly expressed at the presynaptic terminal in the dorsal horn. Consequently, one can make recordings from spinal cord neurons and apply capsaicin to activate presynaptic terminals of TRPV1-expressing nociceptors, which produces an excitatory postsynaptic current in the second order neurons with which TRPV1 positive nociceptors are connected (Yang et al. 1998). In mice, the vast majority of postsynaptic neurons that are synaptically driven by TRPV1-positive nociceptors are located in the superficial dorsal horn, but in the naked mole-rat just as many deep dorsal horn neurons are coupled to capsaicin-sensitive nociceptors as are neurons in superficial layers (Park et al. 2008). This altered connectivity of TRPV1-positive nociceptors potentially explains why capsaicin elicits a robust nocifensive response in mice, but none in the naked mole-rat. Thus, activation of capsaicin-sensitive nociceptors in the naked mole-rat simultaneously activates both superficial and deep dorsal horn neurons. We speculate that reciprocal connections between synaptically driven interneurons in the superficial and deep dorsal horn could under such circumstances negate a pain signal. Interestingly, when SP was introduced into the naked mole-rat, for example through intrathecal injection, capsaicin injected into the paw was newly able to evoke a nocifensive licking response and provoked thermal hyperalgesia (Park et al. 2008). The neurokinin-1 receptor (NK1R) for SP is only present in the superficial dorsal horn of the naked mole-rat spinal cord and thus presumably activation of nociceptors by capsaicin in the presence of spinal SP leads to a stronger activation of superficial layers that may allow gating of a nocifensive response (Fig. 1) (Park et al. 2008). Administration of SP was also shown to “rescue” histamine-induced itch in naked mole-rats (Smith et al. 2010) and evoke a thermal hyperalgesia that is both NMDA receptor- and NK1R-dependent (Brand et al. 2010).

## Absence of acid-induced pain in naked mole-rats

In addition, to capsaicin insensitivity, naked mole-rats also show no pain behavior in response to foot pad injection of acid (Park et al. 2008). Acidic solutions (e.g., pH 3.5 similar acidity to lemon juice) cause stinging pain in humans (Steen and Reeh 1993; Reeh and Steen 1996). Nocifensive responses to acid solutions have been observed throughout the animal kingdom from *C. elegans* (Sambongi et al. 2000), to the rainbow trout *Oncorhynchus mykiss* (Sneddon et al. 2003), mice (Price et al. 2001; Park et al. 2008) and humans (Reeh and Steen 1996; Ugawa et al. 2002; Jones et al. 2004; Schwarz et al. 2017), which makes the lack of acid-response observed in naked mole-rats almost unique (see below). Moreover, whereas capsaicin activates naked mole-rat C-fibers no acid excitation of naked mole-rat C-fibers was observed, in mice all acid activated C-fibers are normally also capsaicin sensitive (Price et al. 2001). Since naked mole-rat nociceptors are not activated by acid it was not surprising that intrathecal application of SP in the naked mole-rat was unable to “rescue” any acid-induced behavior (Park et al. 2008).

Acid, or more specifically protons, can activate sensory neurons by the modulation or activation of several different classes of ion channels including activation of TRPV1, acid-sensing ion channels (ASICs) and proton-sensing G-protein-coupled receptors (GPCR) and inhibition of certain two-pore K^+^ channels (Holzer 2009; Pattison et al. 2019). Direct electrophysiological studies have shown that naked mole-rat DRG neurons display depolarizing currents to proton application, indeed both TRPV1-like and ASIC-like inward currents have been observed (Smith et al. 2011; Schuhmacher et al. 2018) and expression of different ASIC subunits appears to be roughly equivalent between mouse and naked mole-rat sensory neurons (Schuhmacher and Smith 2016). When examining the proton sensitivity of cloned acid-sensitive proteins, naked mole-rat TRPV1, ASIC1a and ASIC1b have a similar proton sensitivity to their mouse orthologues (Smith et al. 2011); however, the ASIC3 subunit is proton insensitive and appears to shift the proton sensitivity when present in heterotrimers, which may alter sensory neuron acid sensitivity to some extent (Schuhmacher et al. 2018). It was thus puzzling that isolated naked mole-rat sensory neurons can be excited by protons, but why did we observe no acid driven action potentials in nociceptors and no pain behavior? The first clue to solving this puzzle came with the observation that protons can potently inhibit voltage-gated sodium channel (Na_V_) subunits (Khan et al. 2006). Voltage-gated sodium channels are necessary for action potential initiation and propagation, as demonstrated by the fact that pathological mutations in some genes encoding Na_V_ subunits cause CIP with no gross alterations in sensory neuron anatomy (Cox et al. 2006; Leipold et al. 2013). Although recent work suggests that loss of function mutations in the *SCN9A* gene encoding Na_V_1.7 do lead to structural changes in nociceptors (McDermott et al. 2019) The Na_V_1.7 subunit is particularly important for action potential initiation and the naked mole-rat gene encodes amino acid variations that when mutated into the human protein considerably enhance proton block of Na_V_1.7 channels at certain pH values that excite nociceptors (Smith et al. 2011; Harms et al. 2017). We also showed that the low pH is a much more potent inhibitor of mechanically evoked nociceptor firing in naked mole-rat nociceptors than in the mouse. Thus our model predicts that at least one major mechanism by which naked mole-rats have evolved acid insensitivity is through highly specific changes in residues that enhance proton block of the Na_V_1.7 channel (Smith et al. 2011). Interestingly, in an ex vivo lumbar splanchnic nerve preparation, protons have been shown to evoke neuronal activation in naked mole-rats (albeit to a lesser extent than in mouse) (Hockley et al. 2020) and this can probably be explained by the fact that Na_V_1.7 is far more important in somatic pain than visceral pain (Hockley et al. 2017).

## Inflammatory pain in naked mole-rats

In addition to changes in acute nociception, a variety of inflammatory stimuli have been used to examine the development of hyperalgesia in naked mole-rats. Formalin is a substance that evokes acute and late phases of nocifensive behaviors, which although occurring in the naked mole-rat are diminished compared to in mice (Park et al. 2008; Eigenbrod et al. 2019). The effects of a variety of analgesics and anti-inflammatory agents have been tested in the formalin model and the results can be summarized as follows: opioid receptor agonists and nefopam decrease both early and late phases of the pain response, whereas paracetamol, naproxen and dexamethasone only the late phase (Karim et al. 1993; Kanui et al. 1993; Towett et al. 2009), i.e., compounds whose mechanism of action is predominantly anti-inflammatory only affect the late phase. There is also evidence that activation of muscarinic receptors, in particular M1 and M4, reduces formalin-induce pain behavior in the naked mole-rat, although the circuitry underlying these effects are poorly understood at present (Dulu et al. 2014; Jørgensen et al. 2016).

**Fig. 2:**
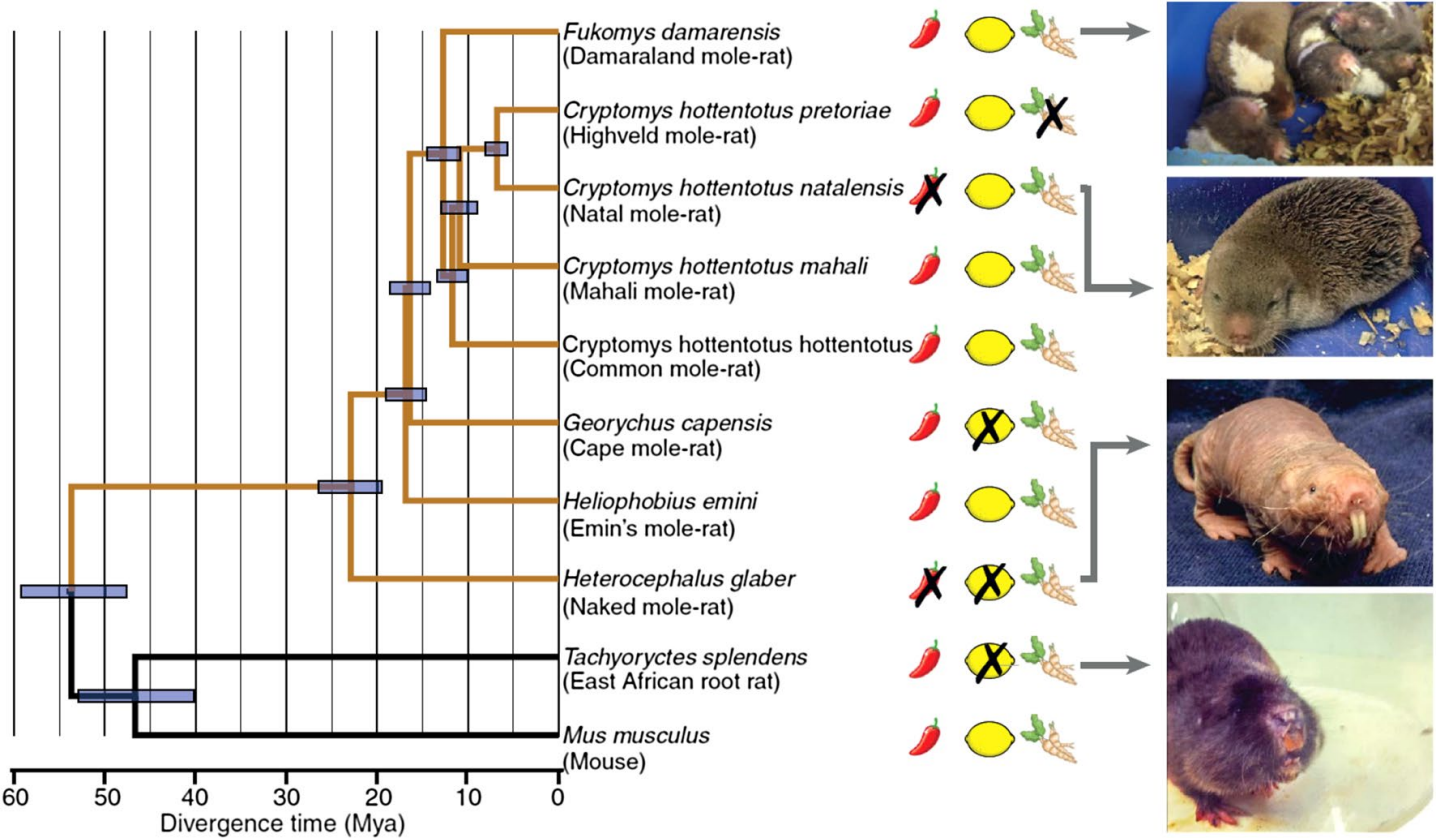
Algogen insensitivities in African rodents. Phylogenetic tree of the studied rodent species, as calculated on the basis of transcriptomic data. Divergence times were calculated on the basis of published and de novo assembled transcriptomes. An “X” indicates an insensitivity to the algogen. Mya, million years ago. [Photo credits (top to bottom): Karlien Debus, Gary Lewin, Jane Reznick] Illustration courtesy of Science

As discussed above, capsaicin fails to evoke thermal hyperalgesia in the naked mole-rat and a similar absence of thermal hyperalgesia was observed following administration of complete Freund’s adjuvant (CFA) and NGF, although mechanical hyperalgesia was evoked by CFA (Park et al. 2008). NGF-induced hyperalgesia is a highly relevant physiological phenomenon observed in rodents and humans (Lewin et al. 1993, 1994, 2014; Petty et al. 1994). Increased NGF in inflammation is causative of chronic hyperalgesia in animals and humans (Lewin et al. 2014). NGF-induced thermal hyperalgesia is a TRPV1-dependent process (Chuang et al. 2001), but as mentioned previously, naked mole-rat sensory neurons respond to capsaicin, which suggests that loss of function in naked mole-rat TRPV1 is unlikely to explain the absence of NGF-induced thermal hyperalgesia. Indeed, subsequent analysis of naked mole-rat TRPV1 demonstrated that it has normal heat, pH, voltage and capsaicin sensitivity (Smith et al. 2011). Naked mole-rat sensory neurons also express the NGF receptor TrkA, but even in cultured DRG neurons NGF fails to induce sensitization of TRPV1 (Park et al. 2008; Omerbašić et al. 2016). However, NGF sensitizes naked mole-rat TRPV1 when expressed in sensory neurons from mice that lack TRPV1 and when co-expressed with rat TrkA in naked mole-rat fibroblasts, results ruling out deficits in naked mole-rat TRPV1 and the naked mole-rat cellular environment, respectively. Indeed experiments using naked mole-rat TrkA and a chimeric TrkA (extracellular rat/intracellular naked mole-rat) further showed that activation of the naked mole-rat TrkA receptor is much less efficient at producing NGF-induced TRPV1 sensitization, thus indicating that the deficit lies in the intracellular domain (Omerbašić et al. 2016). Furthermore, quantitative proteomics demonstrated that NGF activation of chimeric TrkA receptors is less efficient in engaging downstream signaling as shown by reduced abundance of phosphopeptides compared to rat TrkA. Thus the lack of NGF-induced thermal hyperalgesia in naked mole-rats is almost certainly primarily due to TrkA hypofunctionality (Omerbašić et al. 2016). Examination of the naked mole-rat TrkA sequence shows that between 1 and 3 amino acid substitutions that are unique to the naked mole-rat in the kinase domain of TrkA are likely responsible for the hypofunctional signaling observed, both with regard to the absence of NGF-induced thermal hyperalgesia and the loss of cutaneous C-fibers in adulthood (Omerbašić et al. 2016). To confirm the role of changes in the TrkA kinase domain underlying the phenotypes observed in the naked mole-rat, one would ideally make a transgenic naked mole-rat with the mouse TrkA sequence, but this is currently not feasible. However, we have already generated mice with the critical residues from the naked mole-rat reengineered into the mouse genome. Initial results with these mice indeed indicate that they show reduced inflammatory pain phenotypes (unpublished results). Thus, since the naked mole-rat ancestor appeared more than 30 million years ago (see Fig. 2) the mole-rat had already blindly discovered the analgesic potential not only of reduced NGF signaling, but also of modulating sensory specific ion channels like Na_V_1.7. Both NGF signaling and Na_V_1.7 are actively being pursued as analgesic targets in humans with highly promising results (Lewin et al. 2014; Habib et al. 2015; Luiz and Wood 2016; Hefti 2019).

## Algogen insensitivity: a family affair

The extraordinary sensory attributes of the naked mole-rat recently led us to ask whether pain insensitivity is a commonly occurring attribute among African underground dwelling rodents. The naked mole-rat belongs to the family Bathyergidae, a very species rich group of mole-rats that have spread throughout most of Africa. We decided to systematically test algogen sensitivity of 8 mole-rat species and one root rat species from east Africa (*Tachyoryctes splendens*) (Eigenbrod et al. 2019). We used three algogen stimuli, capsaicin, acid (pH 3.5 HCl) and AITC (allyl isothiocyanate), the latter molecule is the active pungent ingredient of mustard oil and wasabi and is a highly electrophilic molecule that is a specific agonist of the transient receptor potential cation channel subfamily A member 1 channel (TRPA1) (Story et al. 2003; Jordt et al. 2004; Bandell et al. 2004; Hinman et al. 2006). So far, not just mammals find AITC and related TRPA1 ligands noxious, but also flies, planaria, reptiles and birds avoid this substance (Kang et al. 2010; Saito et al. 2014; Oda et al. 2016; Arenas et al. 2017). We also showed that naked mole-rats show robust nocifensive behavior after hindpaw injection of AITC (Eigenbrod et al. 2019). Thus, naked mole-rats can display the same nocifensive behaviors as all other rodents so far tested.

Interestingly, we discovered that one additional mole-rat species was also completely insensitive to capsaicin (the Natal mole-rat *Cryptomys hottentotus natalensis* ) and two additional species were insensitive to acid (Cape mole-rat (*Georychus capensis*) and East African root rat) (Eigenbrod et al. 2019). The mechanisms that might underlie the loss of acid- and capsaicin-induced pain in African rodents are discussed below. Probably, most surprisingly we also identified the first case of complete insensitivity to the pungent chemical AITC in the Highveld mole-rat. This is a social African mole-rat that belongs to the *Cryptomys* genus and like other members of the genus tested (Common mole-rat, Mahali mole-rat and Natal mole-rat) all these animals were found to harbor variants in the TRPA1 channel that render the channel much less sensitive to the AITC ligand (Eigenbrod et al. 2019). However, the Highveld mole-rat was even impervious to 100% AITC and normally displays no licking or behavior in the first phase of the formalin test (Eigenbrod et al. 2019). We carried out large-scale transcriptome assemblies from sensory tissues collected from the species tested and quantified changes in gene expression across the phylogenetic tree (Fig. 2). This analysis revealed a single upregulated transcript for *Nalcn* in the Highveld mole-rat that encodes a non-selective leak channel. This transcript encodes a voltage insensitive leak channel (NALCN) that has been implicated as carrying a background leak current in neurons (Lu et al. 2007; Cochet-Bissuel et al. 2014). We speculated that in Highveld sensory neurons the hvNALCN protein is overexpressed to shunt excitability of nociceptors that detect AITC. Evidence for this idea was provided by pretreating Highveld mole-rats with an antagonist of the NALCN channel with the hope that this would rescue AITC behavioral sensitivity. We used verapamil, which has been shown to block NALCN channels (Lu et al. 2007), and remarkably, after systemic treatment, the Highveld mole-rat became transiently sensitive to both AITC and the acute effects of formalin (Eigenbrod et al. 2019). Our data indicate that the Highveld mole-rat has evolved an unusual and effective molecular strategy to become impervious to pain and in principle such a strategy could be harnessed to develop new analgesics. These studies raise the question of what are the evolutionary benefits to losing certain types of pain?

## What selective pressures have driven evolution of pain insensitivity?

Capsaicin insensitivity and inflammatory pain: We now know that at least two species of mole-rat have lost sensitivity to capsaicin during evolution. The Natal mole-rat and naked mole-rat occupy two very different types of habitat and so it is entirely plausible that both species evolved capsaicin tolerance for different reasons. We do know that the TRPV1 receptor is expressed in DRG neurons of both these species and indeed RNAseq profiling revealed that the sensory neurons in both species (and indeed all African mole-rats tested) can be classified at the molecular level in a similar way to those of mice (Usoskin et al. 2015; Eigenbrod et al. 2019). Furthermore, both the Natal and naked mole-rat TRPV1 are activated by capsaicin (Smith et al. 2011; Omerbašić et al. 2016; Eigenbrod et al. 2019). One parsimonious explanation could be that both these species have adapted to eat underground tubers or roots that are rich in vanilloid-like molecules. Unfortunately, there is currently no information available about the vanilloid content of roots in either habitat. The reduced NGF-dependent inflammatory pain displayed by the naked mole-rat may be unique to this species and is strongly associated with reduced TrkA signaling and a substantial loss of C-fiber nociceptors. One extremely strong factor driving naked mole-rat adaptation and possible eusociality is the fact that this species must deal with semi-arid conditions where a lot of work must be expended to search for sparse food resources. Under such conditions, energy efficiency is highly valuable, and it may be that the loss of C-fiber nociceptors may save energy as it is well known that maintaining neurons is energetically costly. Thus loss of nociceptors may be analogous to the loss of hair or thermogenesis in this species which may allow the naked mole-rat to save on energy expenditure under conditions where burrow temperatures have probably remained constant over millions of years (Buffenstein and Yahav 1991; Jarvis et al. 1994; Bennett and Faulkes 2000). Another factor that could contribute to the reduced pain and hyperalgesia seen in naked mole-rats is the robust way in which social rank is reinforced through robust shoving behavior in this species. The queen especially is known to reinforce colony coherence by asserting her dominance physically on colony members (Reeve 1992), it may well be that a very long lifetime of such robust encounters may select for increased pain tolerance.

Acid insensitivity: By testing a large number of African mole-rat species for acid sensitivity we discovered two new instances of acid insensitivity, in the Cape mole-rat (*Georychus capensis*) and the East African root rat (*Tachyoryctes splendens*). The Cape mole-rat and East African root rat are subterranean rodents, but are both solitary animals, thus they may not be exposed to the hypercapnic environments probably experienced by the highly social naked mole-rat. Nevertheless, both the East African root rat and the Cape mole-rat have acquired variants in the domain IV of the Na_V_1.7 channel that convert a normally predominantly positively charged trio of amino acids (KKV, charged +/+/0 in mouse and human) into predominantly negatively charged residues (EKD or EKE, both −/+/− in all subterranean African mole-rats) (Fang et al. 2014; Davies et al. 2015; Eigenbrod et al. 2019). It should be noted that EKE variant has been shown experimentally to increase the proton block of the Na_V_1.7 channel. Interestingly, large-scale sequence analysis of the Nav1.7 channel across the animal kingdom revealed multiple instances of convergent evolution in the same residues of the Na_V_1.7 channel so that, for example, many hibernating species displayed the naked mole-rat-like EKD variant, but closely related, non-hibernating species exhibited variants resembling the mouse or human sequence (Liu et al. 2014). These data provide powerful evidence that the proton sensitivity of the Na_V_1.7 channel is strongly associated with situations of metabolic stress that could produce tissue acidosis (hibernation is associated with metabolic stress). In our recent study we found that the acid-insensitive Cape mole-rat was the only species that has the identical amino acid motif in domain IV of the Na_V_1.7 channel as the naked mole-rat (EKE and not the more common EKD sequence). Indeed this is only the second recorded instance of such a motif, which is so far the only motif that has been shown experimentally to increase the proton block of the channel (Liu et al. 2014; Eigenbrod et al. 2019). Indeed detailed analysis also revealed that a functionally equivalent part of the Na_V_1.7 channel in domain III also has amino acid variants that are unique to the acid-insensitive African mole-rats of the family Bathyergidae (Eigenbrod et al. 2019). The functional importance of such variants, however, remains to be tested. At least in the case of the naked mole-rat it appears to be reasonable to assume that exposure to high levels of carbon dioxide as consequence of living in crowded colonies underground could be one factor driving acid insensitivity. Naked mole-rats show an extraordinary resistance to hypercapnia which does not produce lung edema in this species (Park et al. 2017). We have also shown that naked mole-rats switch to glycolytic metabolism that produces lactic acid when exposed to extreme hypoxia (Park et al. 2017). It thus appears feasible that acid insensitivity evolved as a protective mechanism to not cause pain under metabolically stressful situations. Indeed, in the brain, naked mole-rats appear to have smaller acid-induced currents and decreased acid-induced cell death, suggesting a further protective mechanism (Husson and Smith 2018). It was recently reported that carbon dioxide levels are elevated in naked mole-rat burrows (Holtze et al. 2018), but that the level reached is less than that generally associated with hypercapnia-induced changes in respiration rate. However, the levels of carbon dioxide reached in sleeping chambers where the majority of the colony sleep together remain to be measured. We routinely observe that naked mole-rats assemble in piles of sleeping animals to sleep communally every day multiple times, it is very likely that animals sleeping at the bottom of such piles will be exposed to very low oxygen and high carbon dioxide.

Since the three acid-insensitive species observed here appear to occupy different types of habitats it was all the more surprising that we could also detect multiple common genes that were regulated in the same direction in the DRG of all three species. We could show that all three species showed reduced levels of transcripts encoding ion channels whose activity would be enhanced by protons (Eigenbrod et al. 2019). Examples of such proteins included TWIK1 and ASIC3 (Chatelain et al. 2012; Schuhmacher and Smith 2016). However, we also discovered multiple new genes involved in metabolic processes that may also help all three species cope with acidosis.

AITC insensitivity: Only one species, the Highveld mole-rat was found to be completely insensitive to the pungent electrophilic chemical Allyl isothiocyanate (AITC). Nevertheless other members of the *Cryptomys* genus exhibited a variant of the AITC gated channel TRPA1, which renders the channel less sensitive to this ligand (Eigenbrod et al. 2019). We found that one of the food sources for these mole-rats was the roots of the small matweed (*Guilleminea densa*) and these roots contained substances capable of activating TRPA1 channels (Eigenbrod et al. 2019). It is thus conceivable that during evolution this Genus of mole-rats became less sensitive to the pungency of their main food source. More dramatically, we found that the Highveld mole-rat shares its burrow with a highly aggressive stinging ant species the Natal droptail ant (*Myrmicaria natalensis*). These ants typically bite with their jaws and then inject a venom from abdominal glands into the wound. The venom of the Natal droptail ant contains unusual myrmicarin alkaloids of largely unknown function (Leclercq et al. 2000). We found that extracts from the abdomen of Natal droptail ants produced potent nocifensive responses in several mole-rat species except the Highveld mole-rat. Interestingly, blockade of the NALCN channel in vivo was associated with de novo pain-related behavior in the Highveld mole-rat when exposed to the ant venom (Eigenbrod et al. 2019). Altogether these data suggest that the Highveld mole-rat evolved resistance to the venom of Natal droptail ants in order to be able to exploit regions of Africa avoided by other mole-rat species. We believe that the high expression levels of the NALCN channels allows this species to be impervious to the ant’s venom. Overexpression of NALCN in cells tended to be very toxic as membrane leakiness especially to calcium leads to cell death. It therefore appears likely that the Highveld mole-rat has evolved additional molecular tricks to compensate for increased membrane leakiness associated with high NALCN levels. In mice, recent studies have suggested that upregulation of NALCN mediated currents is associated with increased neuronal excitability in spinal nociceptive circuits (Ford et al. 2018). The biology of NALCN channel is still poorly understood and it is likely that the interaction partners of this channel may govern some of its cellular effects. Nevertheless, the Highveld mole-rat has demonstrated how this channel could be harnessed to shut down neuronal excitability.

In conclusion, studies on African mole-rats have so far revealed novel molecular strategies to ameliorate pain. These animals are a highly valuable resource to discover how evolution can produce novel solutions that will in the end inform us how to best develop therapeutic approaches for a variety of human illnesses as well as pain.

## Abbreviations

AITC: Allyl isothiocyanate
ASIC: Acid-sensing ion channel
CGRP: Calcitonin gene-related peptide
CIP: Congenital insensitivity to pain
DRG: Dorsal root ganglia
GPCR: G-protein coupled receptor
Na_V_: Voltage-gated sodium channel
NALCN: Sodium leak channel non-selective protein
NGF: Nerve growth factor
NK1R: Neurokinin-1 receptor
PRDM12: PRDI-BF1 and RIZ homology domain-containing protein 12
SP: Substance P
TG: Trigeminal ganglion
TRPA1: Transient receptor potential cation channel subfamily A member 1 channel
TRPV1: Transient receptor potential vanilloid 1 channel
TWIK1: Two-pore-domain weakly Inward rectifying K+ channel
